# Bio-Based Ceramic Membranes for Bacteria Removal from Water

**DOI:** 10.3390/membranes12090901

**Published:** 2022-09-19

**Authors:** Pelagie Kamgang-Syapnjeu, Dayirou Njoya, Elie Kamseu, Sebastien Balme, Mikhael Bechelany, Laurence Soussan

**Affiliations:** 1 Laboratory of Applied Inorganic Chemistry, Faculty of Sciences, University of Yaounde 1, Yaounde P.O. Box 812, Cameroon; 2 Laboratory of Materials Analysis, Mission de Promotion des Matériaux Locaux (MIPROMALO), Yaounde P.O. Box 2396, Cameroon; 3 Institut Européen des Membranes, IEM UMR 5635, Univ. Montpellier, ENSCM, CNRS, 34090 Montpellier, France

**Keywords:** bio-based membrane, ceramic membrane, *E. coli* retention

## Abstract

Bio-based ceramic membranes were elaborated from kaolinite clays, coconut husks and eggshells to retain *E. coli* bacteria present in water intended for human consumption. Their characterization and removal performances are investigated in this work. These bio-ceramic membranes were obtained by heating the formulation containing 75% clay, 15% coconut husk and 10% eggshell at 900 °C or 1000 °C, at different temperature rates, to give S1, S2 and S3 materials. Thermogravimetric analysis (TGA) and differential scanning calorimetry (DSC), mercury porosimetry and scanning electron microscopy (SEM) were used to characterize these membranes. Water flux density, bacterial removal and biofouling were also assessed. Water flux density was shown to depend on material porosity. Bacteria retention was 90% (with 1 log-removal) for S1, 80% (with 0.7 log-removal) for S2 and 100% (with 3.3 log-removal) for S3. Membranes S1 and S2 presented reversible biofouling, while no fouling was evidenced for S3 in the tested conditions. This work shows that the best bio-ceramic membrane in terms of bacterial removal and flux density was S3. Its water flux density was 2123 ± 72 L/h/m^2^ at an initial pressure of 0.2 bar. This material is particularly interesting because its production protocol is quite simple, fast and without the addition of chemical additives. Moreover, it can be used to efficiently remove bacteria from drinking water.

## 1. Introduction

Water treatment is one of today’s most important human concerns [1]. With the scarcity and poor quality of water distributed in some regions of the globe, more than 40% of the world’s population will live in water stress conditions by 2050 [1]. Among water pollutants, pathogenic microorganisms are one of the most harmful to human health, especially *E. coli* bacteria. *E. coli* bacteria are the most common indicator of fecal contamination in drinking water [2]. Chemical disinfection is effective against many pathogenic microorganisms (notably bacteria), but the use of physical barriers such as membranes to remove pathogens is particularly interesting since it is efficient and lowers chemical demand [3]. Ceramic membranes are one of the physical barriers that are being increasingly used in water treatment. They are indeed a class of inorganic materials that have specific properties, such as high chemical and thermal stabilities, a good mechanical strength, a wide variety of microstructures, porosity and geometric accessibilities [4]. Nowadays, ceramic membranes have already proven their effectiveness in toxic compound removal from wastewater [5], reduction in turbidity and dyes [6] and retention of *E. coli* bacteria [7].

In order to valorize some household and agricultural wastes, which generally present interesting properties when they are calcined, the elaboration of ceramic membranes has been improved by the introduction of powders from these wastes [4]. This is the case for bio-based ceramic membranes made with ashes from bovine bones [8], rice husk waste [9], sugarcane bagasse [10] and banana peels [11], which create porosity and sometimes reinforce the mechanical resistance of the membranes obtained.

In this work, three bio-based ceramic membranes were fabricated from kaolinite Cameroonian clays, coconut husks and eggshells for bacteria removal. Coconut husks and eggshells are abundant wastes in Cameroon, where they constitute an environmental issue due to their massive accumulation in nature. To the best of our knowledge, such a use of these natural wastes is innovative. These membranes were consolidated by the implementation of two thermal treatments. Thermal programs are indeed intended to have a potential impact on the porosity and the pore size distribution of the membrane [8] and, consequently, a potential impact on its bacterial rejection ability The first thermal treatment was conducted at 1 °C/min up to 500 °C for 2 h, and then 2 °C/min up to 900 °C for 4 h to yield the S1 membrane which has already been reported [12]. The *E. coli* retention by S1 was 90% [12]. The second thermal treatment was conducted at 5 °C/min up to 900 °C or 1000 °C for 2 h to yield, respectively, S2 and S3. The latter treatment aimed to improve the removal performances of the membrane in comparison to S1. The main objective of this work was consequently to elaborate bio-based ceramic membranes that can retain all bacteria (100%) present in water intended for human consumption.

The bio-based ceramic membranes obtained were characterized using TGA/DSC, mercury porosimetry and scanning electron microscopy (SEM). The flux density of water passing across the materials, the bacterial retention, the biofouling and its reversibility were also studied by using a dead-end filtrating system.

## 2. Materials and Methods

### 2.1. Raw Materials

Two kaolinite clays used for this work were sampled locally in two villages located in the Noun department in the West Region of Cameroon at a depth of 1.8 m using hand augers. The first clay was collected in Mayouom and the second one in Koutaba. Coconut husks were collected nearby a coconut market in Edea (Littoral, Cameroon), and eggshells were collected in several cafeterias in Yaounde (Centre, Cameroon).

### 2.2. Preparation of the Bio-Based Ceramic Membranes

Bio-ceramic membranes were fabricated according to the following sequence: (i) preparation of a plastic powder of 75% clay, 15% coconut husk and 10% eggshell; (ii) shaping the plastic powder (physical mixture) by a hydraulic press (FED S. CARVER INC, Menomonee Falls, WI, USA, 53,051) at 3.5 tons to obtain ceramic disks of 4 cm diameter and 2 mm thickness; (iii) drying the bio-based ceramic membranes obtained for 48 h at room temperature to reach maturity; and (iv) consolidation of the dried membranes by thermal treatment at different temperatures, namely 900 °C or 1000 °C, to obtain three bio-based ceramic membranes named S1, S2 and S3 (Table 1). The implemented temperature program consisted of heating the bio-ceramic membranes from room temperature to the final temperature (900 °C and 1000 °C), following two calcined programs: the first is 1 °C/min up to 500 °C for 2 h and then 2 °C/min up to 900 °C for 4 h; the second one is 5 °C/min up to 900 °C or 1000 °C for 2 h.

### 2.3. Bio-Based Ceramic Membranes Characterizations

Different techniques were used to characterize bio-based ceramic membranes. Thermogravimetric analysis (TGA) and differential scanning calorimetry (DSC) were carried out under air with a temperature rise of 5 °C/minup to 1000 °C. α-Al_2_O_3_ was used as a reference. The curves were recorded on a SDT Q600 Simultanee device (TA Instruments)**.** A Fourier transform infrared spectrophotometer (FTIR) Nexus was used to identify the chemical function groups of clays. Scanning electron microscopy (SEM), using a Hitachi S4800 (Hitachi, Issy-les-Moulineaux, France), was used to check the presence of possible defects in the prepared bio-ceramic membranes. TGA/DSC and SEM analyses were carried out once. The porosity and the mean pore diameter were determined using a mercury porosimeter (Auto Pore IV 9500 from Micromeritics, Merignac, France). Porosimetry analysis was conducted twice.

### 2.4. Measurements of Water and PBS Flux Densities

The flux densities of deionized water and phosphate-buffered saline (PBS) at pH 7.0 ± 0.1 were measured by using the dead-end filtrating system already described by [12]. PBS is a solution that was made of NaCl 8.0 g/L, KCl 0.2 g/L, KH_2_PO_4_ 0.24 g/L and Na_2_HPO_4_.12H_2_O 3.58 g/L. The protocol steps were as follows: (i) activation of the pores’ membranes in deionized water during 24 h; (ii) dead-end filtration of 100 mL of ethanol 96% through the filtrating membranes to sterilize them; (iii) rinsing of the materials by first immersing them into sterilize deionized water, and then filtrating 200 mL of deionized water to remove ethanol residues; and (iv) filtration of 250 mL of sterile deionized water first, followed by 250 mL of the sterile PBS, respectively. The filtration time was measured and filtration was reproduced twice. The initial transmembrane pressure was fixed to 0.2 bar.

Flux density J (in L/h/m^2^) was obtained using Equation (1):

(1) $$J=\frac{Q}{S}$$

where Q (L/h) is the filtrated flux and S is the sample surface of the material tested (12.56 cm^2^). The flux rate Q was obtained by dividing the known filtrated volume by the filtration time.

### 2.5. Bacterial Removals

#### 2.5.1. Bacterial Suspension Preparation and Filtration

A non-pathogenic Gram-negative *Escherichia coli* bacterium (K12 DSM 423, from DSMZ, Braunschweig, Germany) was used for the retention tests. The bacterial cultures were prepared from frozen aliquots of *E. coli* stored at −20 °C. Lysogeny broth (LB) Miller and microbiologic agar (from Sigma Aldrich, Saint Quentin-Fallavier, France) were used for the culture medium. The aliquots were inoculated into a fresh LB medium (4% *v/v*) and incubated for 18 h at 30 °C under constant stirring (180 rpm), until the optical density at 600 nm (OD_600nm_) of the bacterial culture reached nearly five (which corresponds to approximately 10^9^ CFU/mL). In these conditions, bacteria were in a stationary phase. Once prepared, the bacterial culture was centrifuged at 4000 rpm for 20 min at 12 °C to remove the culture medium. The bacterial pellets were then resuspended in the same volume of PBS to avoid further bacterial growth, and to stabilize the bacterial concentration while maintaining bacteria viability. The bacterial suspension was then diluted successively by ten in PBS to reach a concentration of about 10^4^ CFU/mL.

A total of 250 mL of the freshly prepared bacterial suspension was then filtrated in dead-end mode on the sterilized and conditioned filtration cell [12]. Each filtration experiment was duplicated.

#### 2.5.2. Bacterial Counting and Assessment of the Bacterial Removals

A conventional plaque assay method was used to enumerate the bacteria in the bacterial suspension and the permeate [12]). Each count was duplicated. The quantification limit was 3 CFU/mL, where CFU means colony-forming unit. Negative controls (i.e. without bacteria) were always run in parallel to check the sterility. The bacterial removal was expressed either in log-removal value (LRV in log) or in a reduction rate (P in %). The LRV was calculated according to Equation (2):

(2) $$\mathrm{LRV}=\log_{10}\left(\frac{A}{B}\right)$$

where A is the number of cultivable bacteria counted in the bacterial suspension that was filtrated, and B is the number of cultivable bacteria counted in the filtrate.

Reduction rate can be calculated by following Equation (3).

(3) $$P=\frac{\left(A-B\right)\times 100}{A}$$

The formula to convert LRV into reduction rate is given by Equation (4).

(4) $$P=\left(1-10^{-\mathrm{LRV}}\right)\times 100$$

### 2.6. Biofouling and Its Mechanical Reversibility

At the end of the filtration of the bacterial suspension, backwashing steps were carried out by returning the filtrating material and first filtrating 300 mL of PBS, and then 250 mL of sterile deionized water. After each backwash, the material was put back in its original position and the flux densities of water were measured according to step (iv) of Section 2.4. The relative loss rate of flux densities was assessed using Equation (5).

(5) $$\mathrm{PF}=\frac{(F_{i}-F_{f})\times 100}{F_{i}}$$

where $F_{i}$ is the initial flux and $F_{f}$ is the final flux, i.e. the flux density measured after backwashing.

The fouling is totally reversible when PF is zero and irreversible (partially or totally) when PF is different from zero.

## 3. Results and Discussion

### 3.1. Characterizations of the Bio-Based Membranes

**Figure 1:**
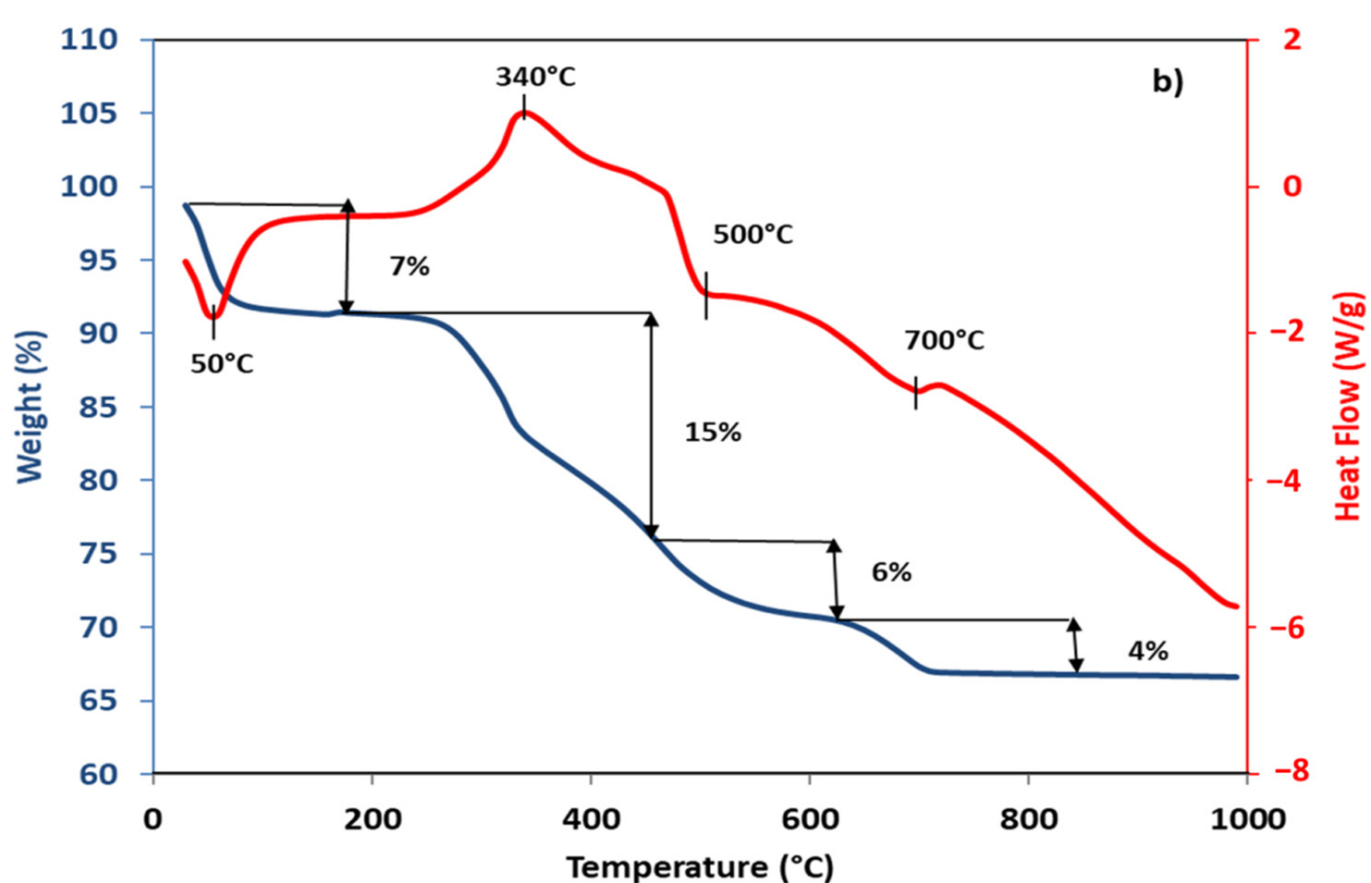
ATG/DSC curves of the formulation used to make all bio-based membranes after sintering.

TGA and DSC curves of the raw plastic powder formulation used to make all bio-based membranes obtained are shown in Figure 1.

At 50 °C, an endothermic peak was observed with a mass loss of 7%, which corresponds to the elimination of free water on the material surface [13,14]. At 340 °C, an exothermic peak was observed with a mass loss of 15%, which corresponds to the decomposition of organic matter from eggshells and coconut husks (cellulose and hemicellulose) [15]. At 500 °C, an endothermic peak was observed with a mass loss of 6%, corresponding to the dihydroxylation of kaolinite into metakaolinite [16,17]. At 700 °C, an endothermic peak was observed with a mass loss of 4%, which corresponds to the transformation of metakaolinite mixed with calcium oxide (due to the decomposition of eggshells) into anorthite [18,19].

Chemical analyses of the three bio-based membranes fabricated showed that the major phase contains aluminum, silicon and calcium. The presence of aluminum and silicon refer to alumina and silica, which can be attributed to mullite and quartz [12]. The presence of calcium refers to calcium oxide which can be attributed to anorthite, as shown by the ATG/DSC curves (Figure 1).

Table 2 shows the porosity and pore diameters of S1, S2 and S3 membranes obtained by using a mercury porosimeter.

Table 2 reveals that the porosities of the bio-based membranes S2 and S3 were almost identical (about 30%), but they were reduced by nearly half in comparison to that of S1 (52%). Their average pore diameters were also almost similar (about 0.06 µm) but were smaller than that of S1 (0.08 µm). The same heating rate of 5 °C/min and the increase in the calcination temperature from 900 °C to 1000 °C (Table 1) thus has no influence on the porosity and the average pore diameter: the S2 membrane calcined at 900 °C and S3 calcined at 1000 °C, exhibiting approximately the same microstructural characteristics. However, these properties are closely linked to the heating rate used. Indeed, the slower the heating rate was (for the S1 membrane: 1 °C/min from 25 °C up to 500 °C during 2 h, and then 2 °C/min up to 900 °C during 4 h, Table 1), the higher the porosity of the material obtained, compared to the one obtained with a rapid temperature raise (for the S2 and S3 membranes: 5 °C/min from 25 °C up to a final temperature of 900 °C or 1000 °C, respectively, during 2 h). This could be attributed to the decomposition rate of the organic matter (decarboxylation) or to the dehydration shown in Figure 1. In fact, the slow decomposition of organic matter between 25 °C and 500 °C is likely to promote the creation of pores in the materials produced (Mouiya et al. 2019; Mohamed et al. 2020), unlike the rapid decomposition of organic matter which would rather reduce the porosity of the material and contribute to the densification of the porous structure during calcination [11,20].

**Figure 2:**
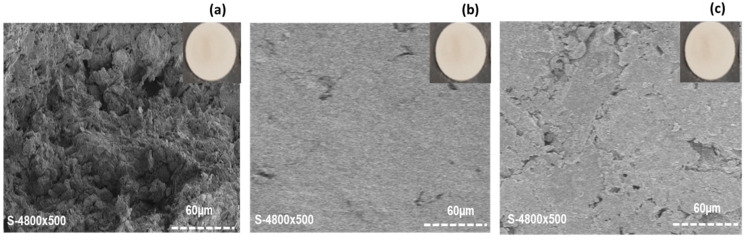
SEM pictures of the bio-based membranes S1 (**a**), S2 (**b**) and S3 (**c**). Pictures of membranes are also given at the top corners.

The morphologies of the different bio-based membranes surfaces were characterized by SEM analysis and the pictures obtained are shown in Figure 2.

Figure 2a–c show the images of the corresponding membranes S1, S2 and S3.

For all membranes, the SEM pictures show that the grains were interconnected with each other, forming a compact structure.

### 3.2. Water and PBS Flux Densities of the Bio-Based Membranes Elaborated

**Figure 3:**
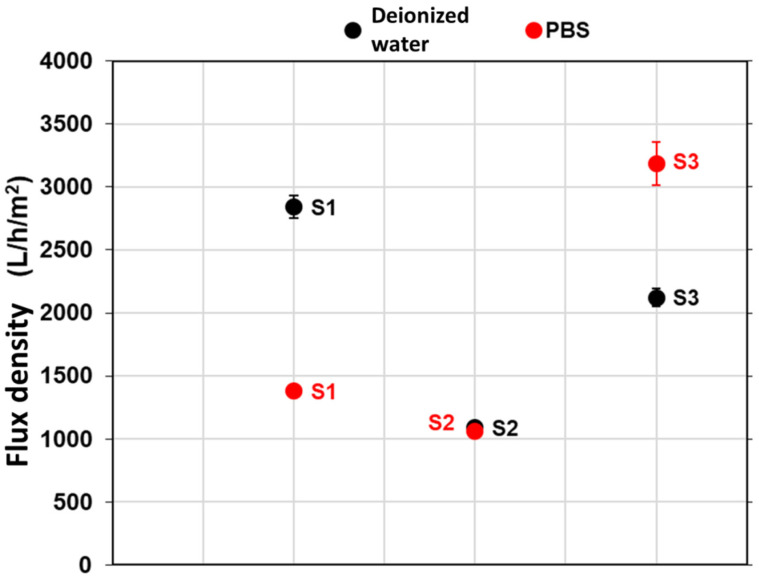
Water and PBS flux densities of the bio-based membranes elaborated.

The water and PBS flux densities measurements are presented by Figure 3 with the associated standard deviations.

Despite similar porosities and mean pore diameters (Table 2), the water flux density obtained for the S2 membrane was 1062 ± 19 L/h/m^2^, while that of the S3 membrane was nearly twice the amount (2123 ± 22 L/h/m^2^). This phenomenon could be due to differences in the material tortuosity, which is a geometric parameter defining the path traveled by a fluid in a membrane, due to the complexity of the porous medium [21,22]. The tortuosity is possibly more marked in the S2 membrane, hence inducing a higher flux resistance through the material and smaller flux values observed (Figure 3). The presence of high tortuosity in the S1 membrane might also explain why its flux density (2843 ± 92 L/h/m^2^) is about the same order of magnitude as the S3 one, despite the porosity of the S1 membrane being significantly higher (52% vs. 30%, Table 2).

It appears from Figure 3 that the S3 membrane had a higher flux value for PBS than for water. This was probably due to the affinity that the latter had towards the cations (Na^+^ and K^+^) present in the PBS. This affinity could rely on the cation exchange capacity of the clays implemented in the S3 membrane, as suggested by Kamgang-syapnjeu et al. (2020) [12]. On the contrary, the PBS flux density measured for the S1 membrane is about twice lower than the one obtained with water (Figure 3). This result could arise from tortuosity or adsorption phenomena [20,23], which might occur in the S1 membrane. Finally, the behavior of the S2 membrane appears to be similar to water and PBS. It was noticed that membranes S2 and S3 obtained for the highest temperature rate (Table 1) did not show any flux reduction when passing from water to PBS filtration.

### 3.3. Bacterial Retention Performances

The retention performances of *E. coli* bacteria by the fabricated bio-based membranes are summarized in Table 3, either expressed in log-removal value (LRV) or reduction rate (P). The results are presented with their standard deviations (n = 2).

On one hand, Table 3 shows that the S2 membrane retained 80% of bacteria with a log-removal of 0.7 log, which is inferior to 1 log that is the minimum retention recognized by the World Health Organization to be representative of a significant retention performance [24]. Therefore, using a rapid sintered temperature with a final temperature of 900 °C (Table 1) significantly reduced the bacterial retention rate compared to the S1 membrane (90% with 1 log-removal). On the other hand, the S3 membrane obtained at a final temperature of 1000 °C, with the same sintering temperature rate as the S2 membrane, retained 100% of the bacteria with a log-removal of 3.3 log.

The high retention rate of the S3 membrane could be due not only to the mean pore diameter which is small (0.06 µm, Table 2), but also to the higher material densification allowed at 1000 °C that might induce a homogeneous distribution of the pore size. At this temperature (1000 °C), the well-crystallized anorthite phase was indeed more present than at 900 °C (S2 membrane, Table 1) [25], and the mullite phase (pseudo) could be better crystallized than at 900 °C, thus contributing to the material densification [26]. These phases have been detected in the raw materials characterizations used to elaborate upon S2 and S3 membranes [12].

This densification might prevent the pores from being deformed under the effect of pressure during filtration (convective force). On the contrary, the S2 membrane (calcined at 900 °C) had smaller pores (0.05 µm mean diameter, Table 2) but retained fewer bacteria (80%), probably due to the deformation of the pores, a result of the pressure during filtration [7,20].

Among other recent studies reporting bacterial retention with ceramic membranes (Table 4), it can be seen that the S3 membrane is one of the best materials. It could be highlighted that this performance was achieved without the addition of any antibacterial agents, such as silver or photocatalytic nanoparticles (like TiO_2_). The optimal temperature program for the bio-based membrane elaboration in terms of bacterial removal is thus the one implemented to obtain the S3 membrane (i.e. 5 °C/min up to 1000 °C during 2 h).

### 3.4. Characterization of the Fouling during Filtration

Fouling of the filtrating material can result in a decrease in the flux density during operation and, therefore, in the process productivity [20]. Fouling was thus characterized in this study. For this, the material was implemented as follows: water filtration, PBS filtration, filtration of the bacterial suspension, backwashing with PBS, PBS filtration, backwashing with water and water filtration. It should be recalled that bacterial suspension contains bacteria and PBS.

**Figure 4:**
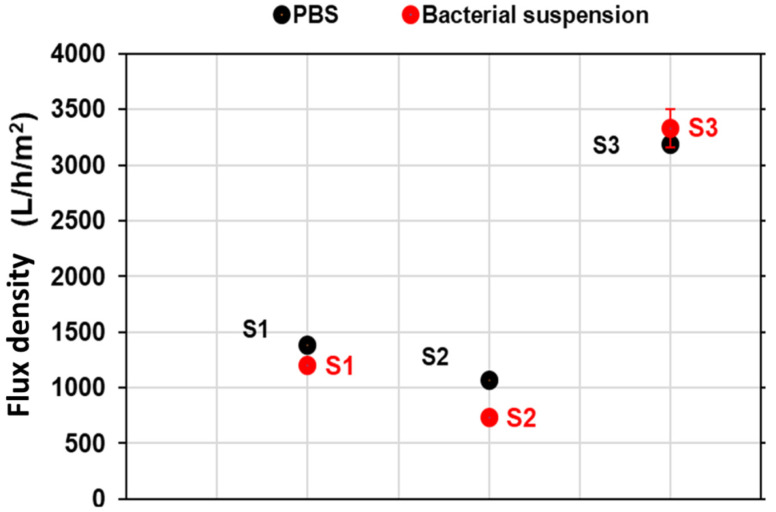
Flux densities with PBS (before filtration of the bacteria) and the bacterial suspension for the bio-based membranes elaborated.

Figure 4 shows the flux densities obtained with the bacterial suspension and the PBS before filtration of the bacteria.

Taking into account the standard deviation, Figure 4 shows that flux densities with the PBS and the bacterial suspension are about the same for the S3 membrane. No bacterial fouling was thus evidenced for the S3 membrane in these conditions. However, the bacterial suspension flux decreased a little for the S1 and S2 membranes compared to the PBS one. This observation is possibly due to the clogging of some membrane pores by the bacteria [7].

Table 5 presents the relative losses of water flux densities measured for the different materials between the operation beginning (i.e. before the bacterial suspension was filtrated) and the backwashing steps following the filtration of the bacterial suspension. When a significant loss of flux density is evidenced, it means that fouling occurred, and that backwashing was not sufficient to recover the initial flux density. In this case, the fouling was assumed to be irreversible in the tested conditions.

Table 5 shows that a small irreversible fouling was observed in the S1 membrane because only 5% of the water flux was lost after backwashes. In other words, 95% of the flux was recovered. The little fouling observed was probably due to bacteria accumulation on the membrane [4,7].

For the S2 and S3 membranes, the relative loss of the water flux was zero, meaning that the water flux densities before and after backwashing were similar. This is consistent with the fact that no flux reduction was evidenced for the S3 membrane (Figure 4). Regarding the S2 membrane, backwashing with water enabled the recovery of its initial flux density; thus, the fouling was totally mechanically reversible (Figure 4).

The absence or small fouling in these membranes could be due to their pore diameters that are too small (about 0.05 µm, Table 2) to allow the *E. coli* (about 0.5–3 μm diameter, Ashbolt 2015) to enter the pores and block them.

The absence of fouling for the S3 membrane is very positive because this material is the best in terms of preparation ease, bacterial retention (100%) and flux density. Its water permeability (10 615 ± 362 L/h.m^2^.bar) was obtained by dividing the flux density by the initial transmembrane pressure (i.e. 0.2 bar), and appears to be part of the highest permeability reported for microfiltration membranes [14,30,31,32].

## 4. Conclusions

Bio-based ceramic membranes were elaborated upon in this work by the thermal treatment of a formulation containing 75% kaolinite clay, 15% coconut husk and 10% eggshell. The results show that the material properties (i.e. porosity, mean pore diameters, tortuosity and exchange ion capacity) were closely linked to the flux density, bacterial retention and fouling.

The work succeeded in obtaining 100% *E. coli* retention (3.3 log-removal) with the bio-based S3 membrane, calcined at 1000 °C. In addition, the water permeability of S3 was among the best ones reported (about 10,000 L/h.m^2^.bar) and no biological fouling was evidenced for this membrane in the conditions tested. The S3 bio-based ceramic membrane is thus a material of high performance in terms of fabrication simplicity, permeability, bacterial retention and biofouling. It can be seriously envisaged in the field of drinking water disinfection.

## Figures and Tables

**Table 1:** Bio-based ceramic membranes elaborated.

Sintering Temperature Programs	Bio-Based Membranes Obtained
1 °C/min up to 500 °C for 2 h and then 2 °C/min up to 900 °C for 4 h	S1
5 °C/min up to 900 °C for 2 h	S2
5 °C/min up to 1000 °C for 2 h	S3

**Table 2:** Porosity and mean pore diameters of the bio-based membranes elaborated.

Bio-Based Membranes	Porosity (%)	Mean Pore Diameters (µm)
S1	51.9 ± 0.1	0.083 ± 0.001
S2	28.2 ± 0.1	0.053 ± 0.002
S3	31.2 ± 0.1	0.060 ± 0.001

**Table 3:** Retention performances of *E. coli* bacteria by the bio-based membranes elaborated.

Bio-Based Membranes	LRV (log)	P (%)
S1	1.0 ± 0.1	90.4 ± 0.2
S2	0.7 ± 0.0	80.2 ± 0.5
S3	3.3 ± 0.3	99.8 ± 0.3

**Table 4:** Recent works reporting bacterial retention with ceramic membranes.

Authors	Materials Used	Retention Performances (%)
[7]	Ceramic membrane (China clay, quartz and calcium carbonate)	90.24% of *E. coli*
[27]	Ceramic-based composite membrane (mullite–carbon nanotubes)	100% of *E. coli* 100% of *S. aureus*
[28]	*Miscanthus* biochar filters Sand filters	1.35 ± 0.27 log of *E. coli* 1.18 ± 0.31 log of *E. coli*
[29]	Fe/TiO_2_ membrane	99.99% of *E. coli*
Our work	Bio-based membrane (clays, coconut husks and eggshells)	100% of *E. coli* (3.3 log)

**Table 5:** Water flux densities measured before filtration of the bacteria and after the backwashes with water.

Membranes	Water Flux (L/h.m^2^)	Water Flux after Backwashing (L/h.m^2^)	Water Loss Rate (PF) (%)
S1	2843 ± 92	2701 ± 32	5.0 ± 0.1
S2	1062 ± 9	1062 ± 19	0
S3	2123 ± 72	2123 ± 22	0

## Data Availability

Not applicable.
